# The design of a new truncated and engineered alpha1-antitrypsin based on theoretical studies: an antiprotease therapeutics for pulmonary diseases

**DOI:** 10.1186/1742-4682-10-36

**Published:** 2013-05-24

**Authors:** Nazanin Pirooznia, Sadegh Hasannia, Seyed Shahriar Arab, Abbas Sahebghadam Lotfi, Mostafa Ghanei, Abbas Shali

**Affiliations:** 1 Department of Biology, Faculty of Sciences, University of Guilan, Rasht, Iran; 2 Department of Biochemistry, School of Biological Sciences, Tarbiat Modares University, Tehran, Iran; 3 National Institute of Genetic Engineering and Biotechnology, Tehran, Iran; 4 Department of Biophysics, School of Biological Sciences, Tarbiat Modares University, Tehran, Iran; 5 Department of Clinical Biochemistry, Faculty of Medical Sciences, Tarbiat Modares University, Tehran, Iran; 6 Research Center for Chemical Injuries, Baqiyatallah University of Medical Sciences, Tehran, Iran

**Keywords:** Alpha 1- antitrypsin, Molecular dynamics (MD) simulation, Neutrophil elastase, Protein docking

## Abstract

Alpha 1- antitrypsin (α1AT) a 54 kDa glycoprotein is a protease inhibitor. In the absence of α1AT, elastase released by lung macrophages, was not inhibited and lead to elastin breakdown and pulmonary problems such as emphysema or COPD. α1AT has three site of N-glycosylation and a characteristic reactive central loop (RCL). As small-scale medicines are preferred for pulmonary drug delivery, in this study α1ATs (1, 2, 3, 4 and 5) were engineered and shortened from the N-terminal region. In order to investigate the effect of different mutations and the deletion of 46 amino acids theoretical studies were performed. Homology modeling was performed to generate the 3D structure of α1ATs. The 10 ns Molecular Dynamic (MD) simulations were carried out to refine the models. Results from MD and protein docking showed that α1AT2 has the highest binding affinity for neutrophil elastase, provided the basis for the experimental phase in which sequences from the five α1AT constructs were inserted into the expression vector pGAPZα and expressed in the yeast *Pichia pastoris*. Although, the α1AT2 construct has the highest inhibitory activity even more that the native construct (α1AT5), results indicated the presence of protease inhibitory function of all the proteins' construct against elastase.

## Introduction

α1AT is a 54 kDa glycoprotein which is a serine protease and broad group of other protease inhibitor. This protein protect the lung from cellular inflammatory enzymes, especially neutrophil (leukocyte) elastase, therefore it is called human neutrophil elastase inhibitor [1-3]. In the absence of α1AT, elastase released from neutrophils, was not inhibited and lead to elastin breakdown and other symptoms like pulmonary problems such as emphysema or COPD in adults [2,3]. Impaired α1AT secretion in liver and accumulation in liver cells also cause cirrhosis in neonatal [4]. Relation between α1AT and numbers of diseases including asthma, rheumatoid arthritis, anterior uveitis and systemic lupus erythematosus suggest that α1AT is not only an anti- inflammation protein but also an immune system regulator [5-8]. α1AT regulates lymphocyte proliferation and cytotoxicity, mediates monocytes and neutrophils function. Also α1AT is suggested as an antiapoptotic factor in lung epithelial cells [9]. Besides it was illustrated that protease-antiprotease imbalance is an important factor in COPD and other pulmonary diseases pathogenesis such as bronchitis. In this process exogenous proteolytic enzymes leads to lung breakdown (because human neutrophil elastase cannot be inhibited by α1AT) [10]. Cigarette smoking is considered as the main cause of COPD which is the fourth leading cause of death in the United States [2]. The oxidation of Met358 in the reactive center loop (RCL) of α1AT leads to a critical decrease in inhibitory capacity of this protein against elastase which cause inactivation in protective function of this protein [11]. It was shown that oxidation sensitivity is a regulatory process, and α1AT inactivation possibly causing lung tissue breakdown in inflammatory site through oxygen radicals released from phagocytes. Therefore, smoking causes oxidation of critical residues (methionine) in α1AT and inactivates this protein. α1AT resistant to oxidation can improve health conditions in COPD patients and also reduction of apoptosis induced by cigarette smoking *in vitro*. α1AT beside antiapoptotic function in *in vitro,* has broad anti inflammatory effects in human. Therefore treatment of CF patients with aerosolized α1AT has been shown to reduce sputum neutrophil numbers, IL-8 concentration and elastase function. The effect of α1AT on IL-8 as a neutrophil-chemoattractant and neutrophil recruitment to lung is also important because neutrophils are considered as the main cause of COPD pathogenesis and clinical effects of α1AT in COPD are obvious [7].

Nowadays the only intravenous infusion of α1AT is available in the world as therapeutic agent which is not the first choice for COPD treatment due to its high cost and low availability [12]. But the aerosol form of α1AT in CF and COPD patients for the prevention of disease progression is not hard to believe [13].

α1AT in its mature has a polypeptide chain consisting of one cystine, two tryptophans and nine methionine residues. α1AT has three site of N-glycosylation which are highly mannosylated because tri- and tetra N-glycans can be attached to aspargine 107 [1].

The oxidation of Met358 in the reactive center loop (RCL) of α1AT leads to a critical decrease in inhibitory capacity of this protein against elastase which cause inactivation in protective function of this protein. It was shown that oxidation sensitivity is a regulatory process, and α1AT inactivation possibly causing lung tissue breakdown in inflammatory site through oxygen radicals released from phagocytes [14,15].

Several mutations within the α1AT genes have been found with the most common point mutations including S and Z mutations with Glu264Val and Glu342Lys substitutions, respectively [16]. FDA has confirmed the utilization of four human plasma derived products of α1AT-Prolastin, Zemaira, Aralast and Glassia as replacement or augmentation therapy. These products which increase the intravenous concentration of α1AT, cost more than 100000$ for each person annually. The infusion form of α1AT has several disadvantages such as: because it has blood origin the risk of viral and non viral contamination is high, also due to the limitation of proteins extracted from blood, huge production is not possible [10,12,17].

The aerosolized α1AT are under study. In the aerosol form, 25%-45% of aerosolized particle reach the respiratory system but in intravenous infusion only 10%-15% of α1AT reach to this site. The aerosolized α1AT not only affect locally its main site of action but also avoid remaining and circulation for a long time in peripheral blood and therefore depress immune response reactions because of long-term use of α1AT [18].

In the other hand, yeasts are effective eukaryotic systems in recombinant protein synthesis in large scale and are able to perform post translational modifications such as glycosylation. In spite of the useful properties of yeasts, these cells are not effective enough for the secretion of high molecular weight glycoproteins [19-22]. Therefore any modification which possibly increases the efficacy and secretion of high molecular weight glycoproteins in yeast can have significant results in protein based medicine products in these cells. With the reduction in size of α1AT beside the preservation of function in production process and increase in the secretion ability of yeast, alteration in glycosylation patterns and elimination of some carbohydrate chains reduce the immunogenicity of secreted α1AT, as a yeast characteristic property is addition of huge mannosylation patterns to secreted proteins.

Huge mannosylation pattern in yeast increases the molecular weight significantly, possibly affects protein activity and also causes difficulty and disturbance in drug deliver to the lung.

Therefore, charbohydrate chains deletion from recombinant α1AT are in the majority of this project. Then, protein length from N-terminal was reduced to produce a smaller molecule which has the same and even higher inhibitory activity as the native α1AT. So, a new α1AT structures were obtained that can be appropriate candidates for pulmonary drug delivery. Therefore, five different α1AT structures with different glycosylation patterns and molecular weights were constructed base of theoretical studies. Beside, each α1AT structures were subjected to three or five different site directed mutagenesis at the same time for both thermal and oxidative inactivation stability. Inhibitory properties of each α1AT constructs were investigated through Elastase Inhibitory Concentration (EIC). Previous studies have shown that the two methionine residues 351 and 358, and in sever conditions methionine 226 are susceptible to oxidation. Oxidation of methionine 358 leads the loss of anti-elastase activity of α1AT. The other methionine residue (Met351) is also as susceptible to oxidation and anti-elastase activity loss as Met358 [23,24].

In this study, site directed mutations in α1AT, produce an active, lower molecular weight, resistant to oxidation and thermal inactivation, with an appropriate half life and unglycosylated protein in *Pichia Pastoris* as a host. In this study we showed that mutations and engineering of the protein molecule, lead to an improvement in inhibitory function. Furthermore, a protein with a less molecular weight and immunogenicity which is suitable for drug packaging and targeting to the lung which is the site of α1AT's action was produce. Molecular dynamic studies showed that deletion of the first alpha-helix from N-terminal sequence have no effect on α1AT inhibitory function. Therefore a reduction of 7 kDa in molecular weight of α1AT, 5 kDa contributing to amino acids weight and approximating 2 kDa to the first carbohydrate chain, was resulted.

## Methods and materials

### α1AT model preparation

Five different protein sequences of α1AT namely α1AT_1_, α1AT_2_, α1AT_3_, α1AT_4 and_ α1AT_5_ were selected (Table 1). Homology modeling of α1AT subtypes was performed using as template the X-ray structure at 2.0 Å resolution of the intact alpha 1-antitrypsin, PDB code file 1QLP. I-TASSER server was applied for the model construction. Structures with the lowest objective function were used for performing molecular dynamic simulation [25].

**Table 1:** Engineered, truncated α1AT structures

**Structure**	**Number of residue**	**Molecular weight**	**Mutation sites**	**N-terminal deletion**
α1AT1	350	39342.3	M351V, M358L, M226L	46 aa
α1AT2	396	44560.8	M351V, M358L, M226L, N83Q, N243Q	_
α1AT3	350	39328.2	M351V, M358L, M226L	46 aa
α1AT4	396	44560.8	M351V, M358L, M226L, N46Q, N243Q	_
α1AT5	396	44532.8	M351V, M358L, M226L	_

### Molecular dynamics simulations

The MD simulations were carried out for 10 ns using GROMACS 4.5.3 software package with the standard GROMOS43a1 force field in the presence of explicit solvent molecules and Na counterions [26,27].

The energy of these systems was minimized using the steepest descent approach realized in the GROMACS package to remove close Van der Waals contacts and to allow formation of hydrogen bonds between water molecules in the periodic box and the protein. A 100 ps MD simulations were performed at the canonical ensemble (NVT) and the temperature was increased from 200 to 300 K. The simulations were continued for another 100 ps at the isobaric-isothermal ensemble (NPT) so that the systems reached equilibration at a constant pressure. The density was stabilized around 0.98 g/cm^3^ during equilibration phase in constant pressure. Finally, a 10 ns MD simulations were performed at the NPT canonical ensemble and the periodic boundary conditions were used in all three dimensions. NVT ensemble simulation was carried out using TIP3P as water models. In all simulations, to keep all hydrogen bonds rigid the SHAKE algorithm was used. The mesh Ewald (PME) summation technique was used to calculate long-range electrostatics interactions; a 14A˚ cutoff for van der Walls interactions, a 12A˚ cutoff for Coulomb interaction with updates every 10 steps, where a time step of 2 fs was considered. In all simulations, the SHAKE algorithm was used to fix hydrogen bond trembling with other atoms. MD simulations were performed in water in a cubic box and 8A° water layer thickness was considered.

Analyses of RMSD, RMSF, solvent accessible surface area, gyration radius, secondary structure, number of hydrogen bonds and dihedral-angle structural distance maps have been carried out using the GROMACS 4.5.3 analysis tools. The first 5 ns of simulation time were discarded to take into account the long system equilibration and all the analyses have been performed on the last 5 ns.

### Molecular docking

The calculations regarding the interaction between neutrophil elastase and α1AT structures, which were obtained by MD simulations, were performed using 3D-Dock program. The structure of neutrophil elastase was obtained from the Protein Data Bank (PDB ID: 2D26). Protein dockings were carried out using MultiDock software package.

Docked structures were constructed using FTDock program. The surface thicknesses of α1ATs were considered as 1.3 Å, and the RPScore program was used to score all known complexes. For each calculation, 100 complexes with the highest score were selected using the MultiDock program. Finally, the complexes at the lowest energy levels were selected and compared with each other.

### Bacteria and *P. pastoris* strains and media

The *E. coli* strain *DH5α* was used for propagation of recombinant plasmids. The *P. pastoris* strain *X-33* (Invitrogen) was used as a host for the protein expression. Recombinant bacteria were cultured in low salt LB broth medium (0.5% (w/v) yeast extract, 1% (w/v) tryptone, 0.5% (w/v) NaCl) supplemented with 25 μg ml-1 of zeocin (Invitrogen). *P. pastoris* was cultured on the following medium: YPDS plates (1% (w/v) yeast extract, 2% (w/v) peptone, 2% (w/v) dextrose, 1 M sorbitol, and 1.5% (w/v) bacteriological agar) supplemented with 100 μg/ml of zeocin.

### Cloning of α1AT in *pGAPZαA* plasmid

For the construction of the expression vector *EcoR*I and *Xba*I sites were introduced respectively at the 5' and 3' end of α1ATs by PCR with specific primers (Table 2). Using DNA from the previous work as template, the resulting 1059 and 1197 bp PCR product was cloned into the pGAPZαA plasmid (Invitrogen, USA).

**Table 2:** Primer sequences

**Name**	**Sequence**	**Mer**
α1AT-Forward	(5'-AAAGAATTCGAGGATCCCCAGGGAGATGC-3')	29
α1AT-Reverse	(5'- TTTTCTAGAGCTTATTTTTGGGTGGATTCACCAC-3')	34
Tα1AT-Forward	(5'- AAAGAATTCAGCACCAATATCTTCTTCTC-3’)	29
Tα1AT-Reverse	(5'- TTTTCTAGAGCTTATTTTTGGGTGGATTCACCAC-3')	34
pGAP –Forward	(5´-GTCCCTATTTCAATCAATTGAA-3´)	22
3́ AOX1	(5´-GCAAATGGCATTCTGACATCC-3´)	21

### *P. pastoris* transformation and selection

*P. pastoris* cells were grown overnight in YPD broth (at 28°C/250 rpm) and prepared for transformation according to the manufacturer’s recommendations (Invitrogen). The recombinant plasmid was linearized with *BspHI* in the *GAP* promoter region and electrotransformed into *P. pastoris X33* electrocompetent cells using a Bio-Rad genepulser apparatus (at 1.5 kV, 25 F, 400 Ω and 8 ms). Zeocin resistant transformants were selected on YPDS agar plates. *P. pastoris* X33 with empty vector was transformed for negative control tests. Integration analysis was confirmed by PCR following with the primers *pGAP* forward and 3'*AOX1* reverse. Correct integration will result in the formation of ~1050-1200 bp PCR products.

### Small scale protein expression

The clones that have given a PCR product of the correct size were used to produce medium-scale cultures. Samples were taken every 24 hours for supernatant analysis. Culture medium was collected after 3 days culture in 250 ml of YPD at 28°C, by centrifugation at 4,000 g for 20 min. The presence of recombinant proteins in the culture supernatant was determined by SDS PAGE, followed by Coomasie staining and Western Blot.

### Large scale protein expression

To obtain large quantities of α1AT fermentation was performed in a 5 L fermentor with a 4 L working volume YPD medium was initially used as the growth medium for α1AT expression. To improve the growth characteristics of the cells in scaled-up fermentation, different media including YPD, YPG and YNB were examined, considering the salts and source of carbon. The rate of air supply and agitation was chosen as high as possible. Therefore, air was supplied at a rate of 10 l/min and the agitation rate was set at 400 rpm under this scale.

### SDS-PAGE and silver staining

The cells were removed and the supernatant was precipitated by using 100% Aceton solution. After drying, the pellet was resuspended in loading buffer (10% w/v SDS, 10 nM β-mercaptoethanol, 20% v/v Glycerol, 0.2 M Tris–HCl, pH 6.8, 0.05% Bromophenol blue w/v), heated for 10 min, in boiling water and electrophoresed at 12% SDS-PAGE/100 V along with molecular weight marker (Fermentas). Gel was stained with silver nitrate according to Celis and coworkers [28].

### Western blot analysis

Protein samples resolved on SDS-PAGE, were electro-blotted to Polyvinylidene Difluoride (PVDF) membrane (Millipore) in transferring buffer (0.025 M Tris, 0.19 M glycine, and 20% (v/v) methanol) overnight at 20 V/4°C. The membrane was treated with PBS-T-BSA (PBS, 0.1% (v/v) Tween 20, 1% (w/v) BSA) for 2 hours to block binding sites. After washing step, membrane was reacted with 1000-fold diluted goat anti-human alpha-1 antitrypsin polyclonal antibody, conjugated with HRP (Abcam) for 3 hr. To eliminate non specific reactions, a supernatant of non- recombinant X-33 culture treated side by side accordingly as negative control. Subsequently, protein bands reacted positively, were visualized at the presence of 4-chloro1-naphtol substrate in PBS.

### Inhibitory activity assay

Elastase activity was measured by EnzChek® Elastase Assay Kit (Molecular Probes, Inc.) according to the manufacturer’s recommendations. The EnzChek kit contains DQ™ elastin soluble bovine neck ligament elastin that has been labeled with BODIPY® FL dye such that the conjugate’s fluorescence is quenched. The non-fluorescent substrate can be digested by elastase or other proteases to yield highly fluorescent fragments. The presence of an inhibitor such as α1AT blocks the substrate digestion hence subsequent fluorescent emission. The resulting change in fluorescence level was monitored using a standard fluorometer (Hitachi F-3010) with a maximum absorption at 505 nm and a maximum fluorescence emission at 515 nm. Commercial human α1AT was used as a positive control and the reaction buffer and the supernatant from non-recombinant *P. pastoris* (X-33 strain) culture as negative controls.

## Results

### Molecular dynamics simulation

#### The root-mean-square deviation (RMSD)

**Figure 1:**
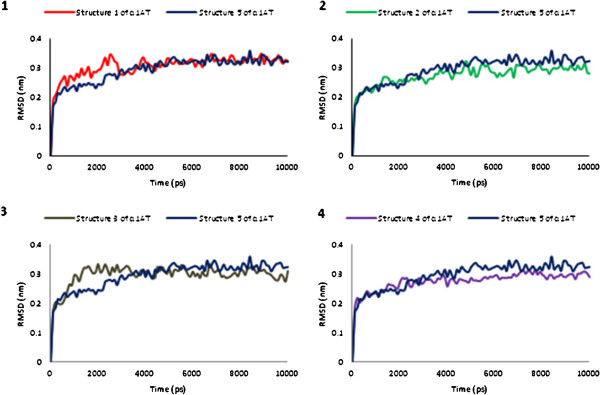
RMSD value of the backbone atoms of each α1AT structures in respect to the reference structure (α1AT5, blue) during 10 ns total simulation time.

One of the most frequently used measures of assessing the stability of an MD simulation over the course of time is the RMSD of the backbone atoms relative to the starting structure during the MD production phase. Although the RMSDs reach a stable value within the first ns, all the analyses have been carried out discarding the first three nanoseconds, i.e. over the last seven nanoseconds (Figure 1). This was done to guarantee an investigation over a well thermalized system.

#### Gyration radius

**Figure 2:**
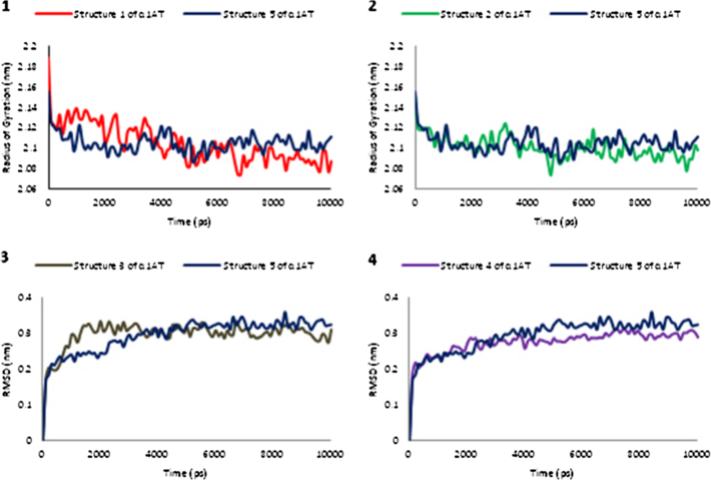
Radius of gyration as a function of time with respect to the starting structures during the MD simulations is shown for each α1AT structures in respect to the reference structure (α1AT5, blue).

To determine the compactness of the chains during the simulations, the time-dependent squared radius of gyration, *R*g ^2^ (*t*) was determined. The radius of gyration gives an estimate of the characteristic volume of a globular polymer, which is inversely related to its compactness (Figure 2).

#### Secondary structure comparison

The analysis of the secondary structures, carried out with the program DSSP by sampling trajectories every 5 ps. Results indicates that the two proteins have comparable secondary structure regions. As shown in Table 3 no difference is observed in the structure of these α1AT mutants (Table 3).

**Table 3:** Secondary structure evolution of the five α1AT structures with simulation time

**Secondary structure of α1AT**	**1**	**3**	**5**	**2**	**4**
**β sheet + turn**	13.8328	13.8328	13.7837	13.7837	13.7837
**Coil**	72.6224	72.6224	72.7027	72.7027	72.7027
**β sheet**	12.1037	12.1037	12.1621	12.1621	12.1621
**Bend**	11.8155	11.8155	11.6216	11.6216	11.6216
**Turn**	1.7291	1.7291	1.6216	1.6216	1.6216
**3-Helix**	1.7291	1.7291	1.8918	1.8918	1.8918

#### Total energy changes

**Figure 3:**
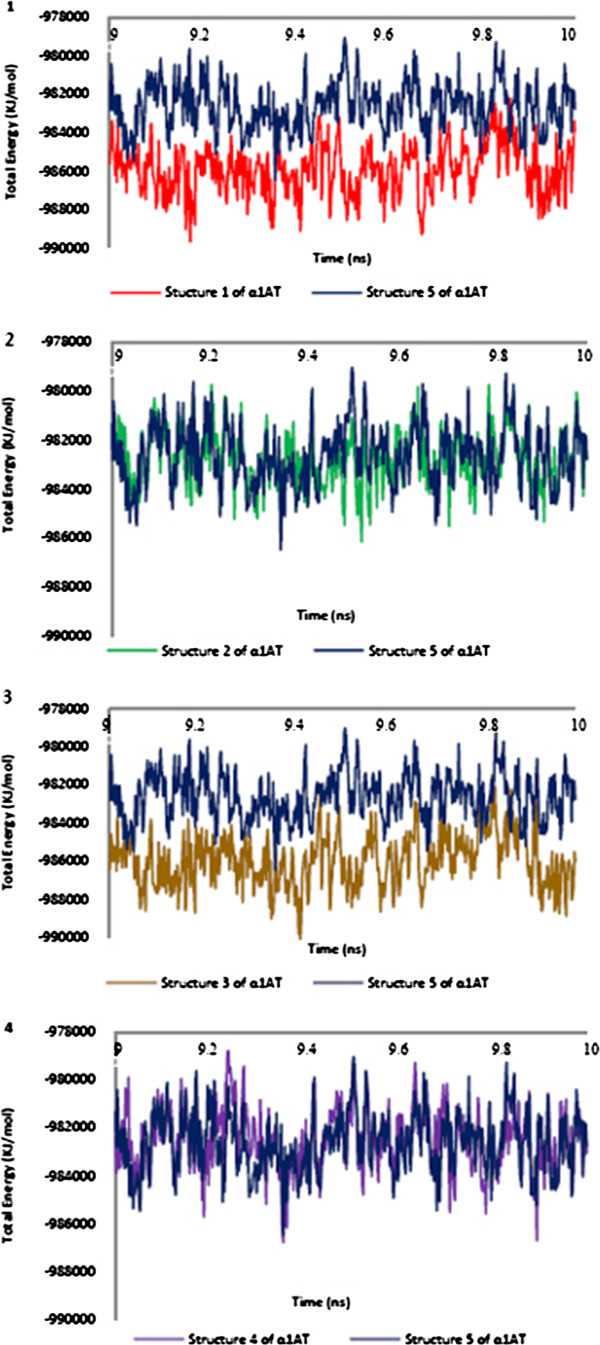
Plots of total energy versus time over the molecular dynamics trajectory for each α1AT structures in comparison to the reference structure (α1AT5, blue).

Total energy changes during 10 ns of simulation time at the equilibrium phase under NPT conditions were carried out for the certitude of simulation stability (Figure 3). As illustrated in Figure, the system’s total energy at the equilibrium phase under fixed pressure conditions after 20 ps for all simulations remains constant.

#### Temperature and density

**Figure 4:**
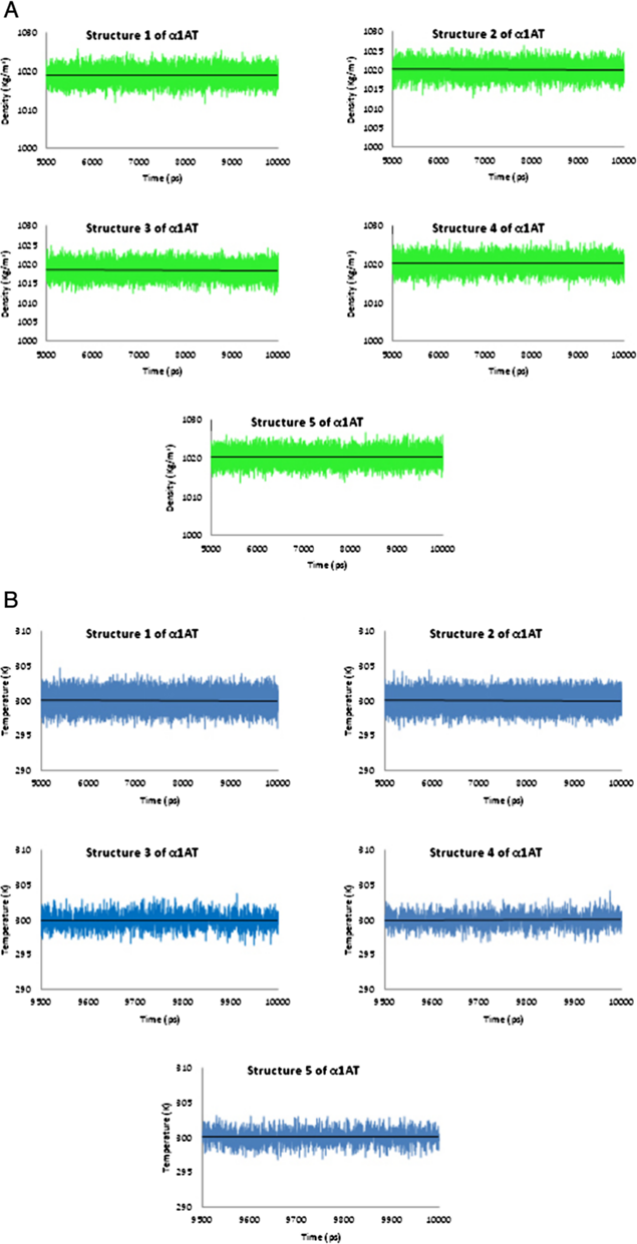
Plot of the system A) density and B) temperature during the 10 ns simulations of α1AT structures.

The temperature and the densities for all α1ATs were 300 K and 1.00 gr/cm^3^, respectively that ultimately became fixed and stable, representing further proof of simulation stability (Figure 4).

#### Structural fluctuations

**Figure 5:**
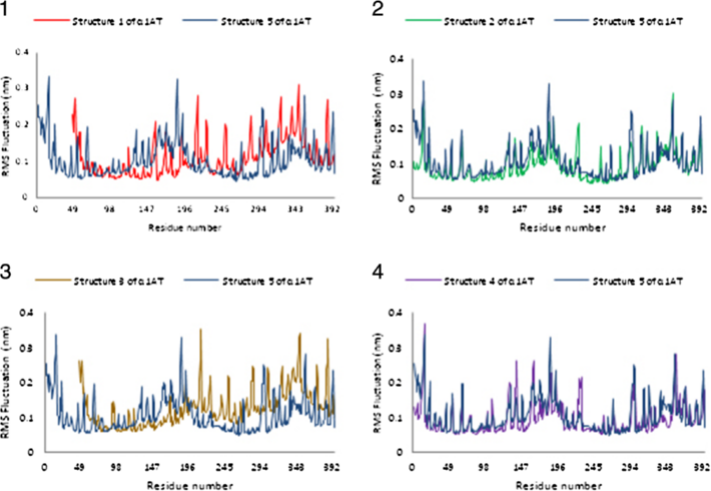
Root mean square fluctuation (RMSF) as a function of residue number for α1AT structures in respect to the reference structure (α1AT5, blue).

Figure 5 shows the C^α^ root mean square fluctuations (RMSF) of α1AT structures in comparison to α1AT structure 5 as a reference. The two α1AT structures 2 and 4 show fewer fluctuations. As revealed in Figure 5, simulation of five structures indicate that a large part of residues is characterized by fluctuations not higher than 2.0 Å, apart from the regions of the C and N-terminal tails. Therefore, the obtained structures from the simulation were optimal and the different structural regions affected each other. Hence, in each simulation, systems have reached to equilibrium.

### Accessible surface area

**Figure 6:**
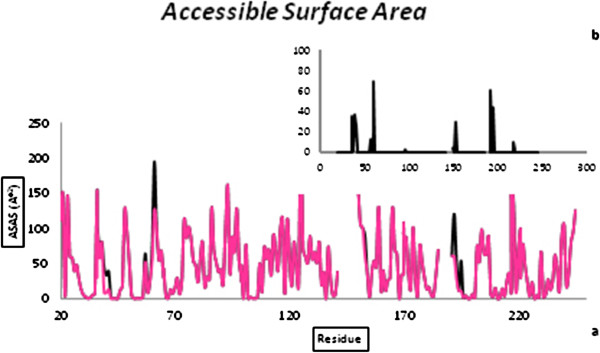
Comparison of accessible surface area of neutrophil elastase in the free and complex form.

The accessible surface area (ASA) was computed considering contribution of side chain atoms and hydrophobic and hydrophilic contributions to the total ASA were calculated during the simulation time (Figure 6).

### Protein docking

In each simulation 100 complexes were made, and complexes with the most binding affinity, which represent the best interaction in complexes, were selected (Table 4).

**Table 4:** Docking energy of α1AT structures and neutrophil elastase

**Structure**	**Energy value**
**α1AT1-Neutrophil Elastase Interaction**	**−41.03 Kcal/mol**
**α1AT2-Neutrophil Elastase Interaction**	−**46.42 Kcal/mol**
**α1AT3-Neutrophil Elastase Interaction**	−**40.37 Kcal/mol**
**α1AT4-Neutrophil Elastase Interaction**	−**41.93 Kcal/mol**
**α1AT5-Neutrophil Elastas Interaction**	−**41.72 Kcal/mol**

### Experimental studies

#### Integration analysis

**Figure 7:**
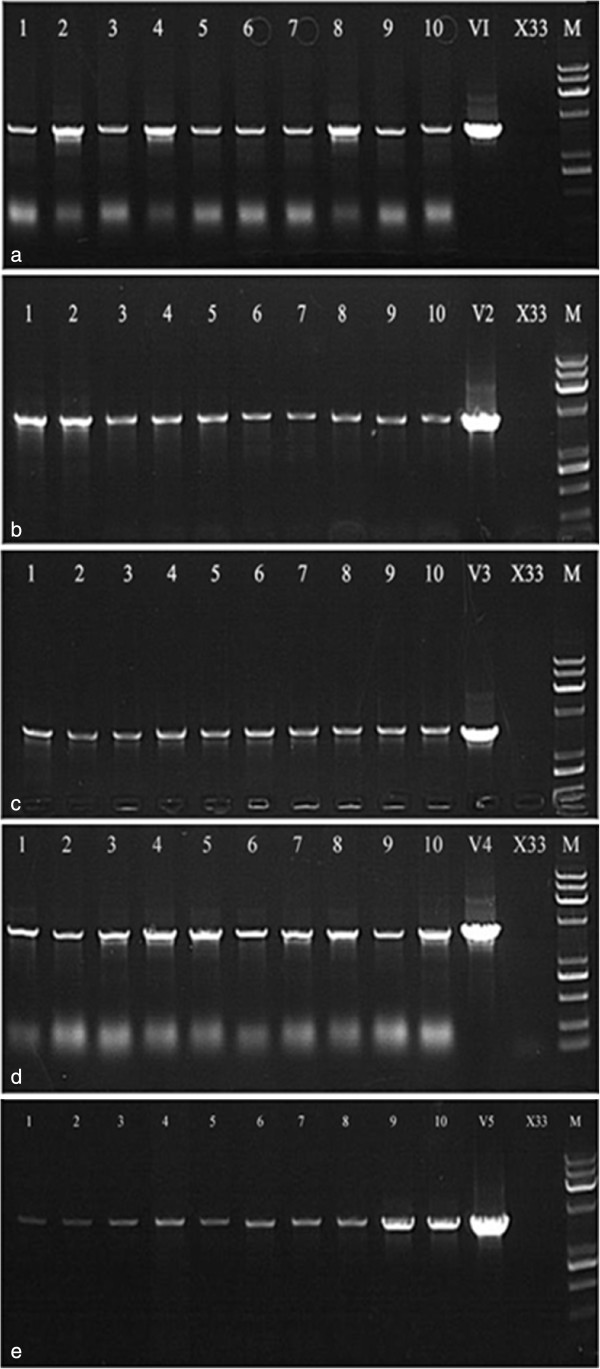
**PCR colony screening was performed using plasmid-specific primers (pGAP forward and 3'AOX) to determine positive clones.** PCR screening of 10 *Pichia pastoris* strains transformed with pGAPZαA/α1AT plasmid. The presence of the specific α1AT −1600 bp PCR amplicon was detected by 1% agarose gel electrophoresis (ethidium bromide staining), confirming the insertion of pGAPZ αA/α1AT plasmid to yeast genome. Lane 1–10: positive colonies of α1AT_1-5_ constructs, V1-5: PCR positive control (pGAPZ αA plasmid) and X33: PCR negative control, M: molecular markers.

Ten zeocin-resistant colonies of each α1AT structures were selected. The integration of α1AT gene into the *P. pastoris* genome is verified by PCR. As shown in Figure 7, PCR products were separated on a 1% agarose gel and stained with ethidium bromide. All PCR fragments have the expected ~1600 bp sizes.

#### SDS page

**Figure 8:**
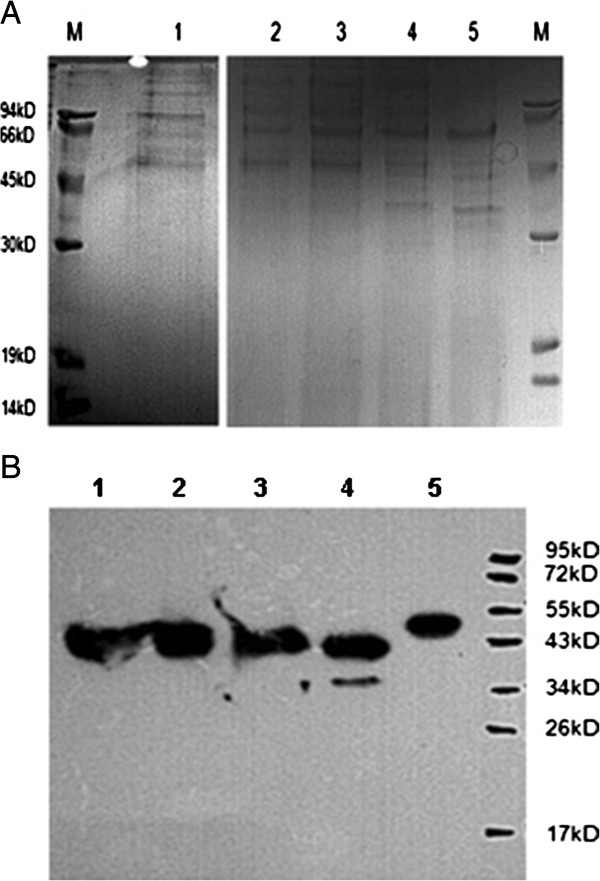
**Expression analysis of constructed vectors containing different α1ATs sequences by SDS–PAGE and western-blot.** (**A**) 72 h after induction, supernatants were electrophoresed in 12% SDS–PAGE and stained by Coomassie blue as follows: lane M, broad-range molecular weight marker; lanes 1, 2, 3, 4 and 5 supernatant samples of α1AT 1, 2, 3, 4 and 5, respectively. (**B**) Proteins were transferred onto a PVDF membrane and identified by immunoblotting using primary and secondary commercial antibodies as follows: lane M, schematic representation of the broad-range marker; lane 1, 2, 3, 4 and 5, supernatant of 72 h inducing culture of α1AT 1, 2, 3,4 and 5, respectively, which treated rabbit anti-AAT polyclonal antibody and then secondary Goat anti-rabbit polyclonal antibody.

The culture expressing native and truncated α1AT was harvested at the following time intervals: 0, 12, 24, 48, 60, 72 and 96 hrs. The supernatant from each culture sample was examined using SDS-PAGE in order to determine the native and truncated-engineered α1ATs expression levels. The 72 hrs time period represented the best time for harvesting in comparison to non-recombinant X-33 culture (Figure 8).

#### Western blot

To confirm the identity of truncated-engineered proteins, proteins were reacted with α1AT antibody in a western blot analysis. The recombinant α1AT was identified as two distinct bands Western blotting using goat polyclonal to alpha-1 antitrypsin for native and truncated-engineered α1ATs is shown in Figure 8, where the native and engineered proteins are detected in both Western blots.

#### Elastase Inhibitory Capacity (EIC)

**Figure 9:**
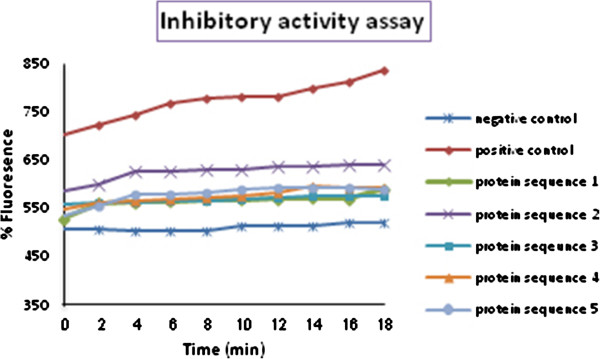
**Elastase inhibitory assay of the five α1AT structures.** α1AT and modified derivatives were incubated with a natural target of α1AT and monitored over 20 min. The substrate is a BODIPY-FL-labeled DQ elastin conjugate that is highly labeled so that the fluorescence signal is quenched until enzymatic digestion yields highly fluorescent fragments. Since DQ elastin is digested by elastase, the inhibition of elastase activity by α1AT can be determined.

The inhibitory function of native and truncated α1AT showed no significant difference and both α1ATs were found to be functional (Figure 9).

## Discussion

Alpha 1- antitrypsin (α1AT) is a 54 kDa glycoprotein which is a serine protease and broad group of other protease inhibitor. This glycoprotein contains 394 amino acids and three glycosylation sites. α1AT protect lung from neutrophile elastase in inflammation or infection therefore it is called neutrophile elastase inhibitor [1,17]. The oxidation of Met358 in the RCL leads to a critical decrease in inhibitory capacity of this protein against elastase which cause inactivation in protective function of this protein. It was shown that oxidation sensitivity is a regulatory process, and α1AT inactivation possibly causing lung tissue breakdown in inflammatory site through oxygen radicals released from phagocytes. Therefore, smoking causes oxidation of critical residues (methionine) in α1AT and inactivates this protein [14,15]. α1AT resistant to oxidation can improve health conditions in COPD patients and also reduction of apoptosis induced by cigarette smoking *in vitro*. The absence or inefficient function of α1AT in the lungs leads to uncontrolled function of elastase and elastin breakdown, resulting in respiratory problems such as COPD and emphysema [3-5]. Association between α1AT and a number of diseases including asthma, rheumatoid arthritis, anterior uveitis and systemic lupus erythematosus suggests that α1AT is not only an anti-inflammatory protein but also an immune system regulator [8-10]. α1AT regulates lymphocyte proliferation and cytotoxicity, mediates monocyte and neutrophil functions. Besides, researchers have shown that the protease-antiprotease imbalance is an important factor in the pathogenesis of COPD and other pulmonary diseases, such as bronchitis. COPD is one of the most important causes of irreversible lung damage and thus the fourth most common cause of death in the U.S. In this process, exogenous proteolytic enzymes lead to lung damage. Besides different physiological roles of α1AT including the control of insulin secretion, antiprotease activity, protecting β-cells against cytokine-induced apoptosis, acting as an anti-inflammation compound [14-16], it is also regarded as an antiapoptotic factor in lung epithelial cells [11]. A recent study has shown that α1AT can slow down the loss of insulin producing cells in diabetic patients who are at the early stages of disease. This study which is in phase ІІ of clinical trials demonstrates the effect of α1AT in preservation of β-cells' function [18,19]. Therefore, only with appropriate and adequate concentrations of α1AT the lungs' correct function can be maintained.

One of the treatment strategies for optimum function of α1AT in inflammation is the replacement therapy using intravenous infusion. In the infusion form only 10%-15% of α1AT reach the target organ. Another possible treatment strategy is by airway delivery. In this form of treatment not only the aerosolized α1AT reach directly the target organ, but also prevent accumulation of excess drug in blood and therefore much lower amount of drug is require [18,29]. Furthermore, in addition to the development and optimization of recombinant form of α1AT, efforts on packaging α1AT in micro and nanoparticles for pulmonary delivery are also under investigations.

During drug delivery, especially protein-based ones, the size of the protein has the major role with regard to its packaging and delivery. In other words, the smaller the protein size, its packaging, stability and delivery becomes more convenient. Protein length from N-terminal was reduced to produce a smaller molecule which has the same and even higher inhibitory activity as the native α1AT. Previous studies have shown that the two methionine residues 351 and 358, and in sever conditions methionine 226 are susceptible to oxidation. Oxidation of methionine 358 leads the loss of anti-elastase activity of α1AT. The other methionine residue (Met351) is also as susceptible to oxidation and anti-elastase activity loss as Met358 [23,24].

Therefore, five different α1AT structures with different glycosylation patterns and molecular weights were constructed base of theoretical studies. Beside, each α1AT structures were subjected to three or five different site directed mutagenesis at the same time for both thermal and oxidative inactivation stability. Inhibitory properties of each α1AT constructs were investigated through Elastase Inhibitory Concentration (EIC).

In this study, site directed mutations in α1AT, produce an active, lower molecular weight, resistant to oxidation and thermal inactivation, with an appropriate half life and unglycosylated protein in *Pichia Pastoris* as a host. In this study we showed that mutations and engineering of the protein molecule, lead to an improvement in inhibitory function. Furthermore, a protein with a less molecular weight and immunogenicity which is suitable for drug packaging and targeting to the lung which is the site of α1AT's action was produce. Molecular dynamic studies showed that deletion of the first alpha-helix from N-terminal sequence have no effect on α1AT inhibitory function. Therefore a reduction of 7 kDa in molecular weight of α1AT, 5 kDa contributing to amino acids weight and approximating 2 kDa to the first carbohydrate chain, was resulted.

Before entering the experimental phase of studies, the effect of eliminating the first 46 amino acids from the N-terminal region, on protein structure was investigated using MD simulation. The native and truncated structures were modeled and the results of the theoretical studies showed no meaningful differences between the properties of these proteins. Results from the dynamic simulations of the molecular models and their interactions with neutrophil elastase showed different interaction energy. The docking energy for the α1AT 2 is less negative which shows better interaction with neutrophil elastase. These findings provided the basis for the experimental phase of the study in which sequences from the five α1AT constructs were inserted into the expression vector pGAPZα. α1ATs were expressed in the yeast *Pichia pastoris* (*P. pastoris*) in a secretary manner and under GAP promoter transformed into *Pichia pastoris*. Thus, experimental studies were carried out to produce recombinant proteins. The level of expression and the functions of the native and truncated proteins were compared. Protein inhibitory activity was investigated through EIC (elastase inhibitory capacity) Results show that all five constructs have a good elastase inhibitory function, although, α1AT 2 has the highest inhibitory activity even more than the native α1AT.

In conclusion, a recombinant and truncated protein with oxidative resistance and without the two main glycosylation chains were obtained which do not effect the inhibitory activity of the α1AT2. The results have important influence in pulmonary drug delivery.

## Competing interests

The authors declare that they have no competing interests.

## Authors’ contributions

NP wrote this manuscript and carried out most of the experiments. SH was main supervisor of this research in Tarbiat Modares University and National Institute of Genetic Engineering and Biotechnology. SSA provided guidance and assistance in modelling and simulation. Professor ASL from Tarbiat Modares University and National Institute of Genetic Engineering and Biotechnology advised on experimental part. Professor MG from Baqiyatallah University of Medical Sciences contributed gave us valuable guidance to improve this work. AS provided assistance in the data analysis. All authors read and approved the final manuscript.
